# Validity and Reproducibility of Food Group Intakes in a Self-administered Food Frequency Questionnaire for Genomic and Omics Research: The Tohoku Medical Megabank Project

**DOI:** 10.2188/jea.JE20240064

**Published:** 2025-03-05

**Authors:** Keiko Murakami, Junko Ishihara, Ribeka Takachi, Shiori Sugawara, Misato Aizawa, Ippei Takahashi, Taku Obara, Mami Ishikuro, Aoi Noda, Mako Ogino, Yuchie Hoshina, Kumiko Kito, Misako Nakadate, Sachiko Maruya, Tomoka Matsuno, Yudai Yonezawa, Takahiro Yamashita, Shigenori Suzuki, Masayuki Yamamoto, Shinichi Kuriyama

**Affiliations:** 1 Tohoku Medical Megabank Organization, Tohoku University, Sendai, Japan; 2 Graduate School of Environmental Health, Azabu University, Kanagawa, Japan; 3 Nara Women’s University Graduate School of Humanities and Sciences, Nara, Japan; 4 Department of Health and Nutrition, Sendai Shirayuri Women’s College, Sendai, Japan; 5 Graduate School of Medicine, Tohoku University, Sendai, Japan; 6 Tohoku University Hospital, Sendai, Japan; 7 Department of Nutrition, Sendai Seiyo Gakuin College, Sendai, Japan; 8 Innovation Division, Kagome Co., Ltd., Tochigi, Japan; 9 International Research Institute of Disaster Science, Tohoku University, Sendai, Japan

**Keywords:** food frequency questionnaire, genomic and omics research, Japan, reproducibility, validity

## Abstract

**Background:**

The Tohoku Medical Megabank Project (TMM) was established to realize personalized healthcare and medicine using genomic and omics data. This study evaluated the validity and reproducibility of food group intakes derived from a self-administered food frequency questionnaire (TMM-FFQ) that included the response option “constitutionally unable to eat/drink it” among community-dwelling Japanese adults.

**Methods:**

Participants comprised 89 men and 124 women aged ≥20 years from Miyagi Prefecture. Participants completed weighed food records (WFRs) for 3 consecutive days per season as reference intake and FFQs in 2019 (FFQ1) and 2021 (FFQ3). Spearman’s rank correlation coefficients (CCs) were calculated for correlations between food group intakes estimated from the 12-day WFR and FFQ3 (validity), and for correlations between those estimated from the FFQ1 and FFQ3 (reproducibility). Cross-classification according to quintiles using FFQ and WFR data was also performed.

**Results:**

The percentage of participants who chose the “constitutionally unable to eat/drink it” option was non-negligible for some food groups. In the validity analysis, CCs were >0.40 for many food groups; the median across 21 food groups was 0.49 in men and 0.45 in women. The median percentages of cross-classification into exact plus adjacent quintiles were 73.0% in men and 66.9% in women. In the reproducibility analysis, CCs were >0.50 for many food groups; the median across 21 food groups was 0.60 in men and 0.51 in women.

**Conclusion:**

The validity of the TMM-FFQ compared with 12-day WFR and the reproducibility of the TMM-FFQ were reasonable for food groups in the TMM cohort studies.

## INTRODUCTION

Obtaining accurate measures of individual dietary intake is an important issue in the analysis and evaluation of results from epidemiological studies on the association between diet and disease. Many epidemiological studies examining diet–disease associations, such as large-scale prospective cohort studies, typically assess long-term diet and rank individuals using specific dietary intake using food frequency questionnaires (FFQs).^1^ Numerous FFQs have been developed tailored to the characteristics of different study populations because food culture and dietary habits vary by population.^2^^,^^3^ However, FFQs do not necessarily evaluate true dietary intake. Therefore, the validity and reproducibility of each FFQ should be assessed.

The Tohoku Medical Megabank Project (TMM) is conducted by Tohoku University Tohoku Medical Megabank Organization (ToMMo) and Iwate Medical University Iwate Tohoku Medical Megabank Organization. The TMM was established to promote reconstruction of the Tohoku region and to address medical problems in the aftermath of the Great East Japan Earthquake and resulting tsunami that occurred on March 11, 2011.^4^ During the process of rebuilding the community medical system, the TMM has been developing a basic infrastructure comprising genome and cohort information to facilitate precision medicine and personalized healthcare.^5^^–^^7^ The TMM has initiated two prospective cohort studies including genome and omics investigation in Miyagi and Iwate Prefectures: a population-based adult cohort study, the TMM Community-Based Cohort Study (TMM CommCohort Study),^8^ and a birth and three-generation cohort study, the TMM Birth and Three-Generation Cohort Study (TMM BirThree Cohort Study), which includes fetuses and their parents, siblings, grandparents, and extended family members.^9^ These cohort studies included a self-administered FFQ (TMM-FFQ) for adults.^10^^–^^16^ One of the unique aspects of the TMM-FFQ is the inclusion of the response option “constitutionally unable to eat or drink it” for individual food and drink items. A person’s constitution is their overall disposition, which is created by interactions between genetic and environmental factors. The response option “constitutionally unable to eat or drink it” assesses the inability to consume a particular food or drink because of a physiological reaction to it, such as sickness, nausea, or allergy. Given the importance of the contribution of genetics to dietary behavior and nutrient metabolism,^17^^–^^19^ this response option should be included in developing personalized nutrition strategies using genetic data. However, to the best of our knowledge, the validity and reproducibility of FFQs that include this response option have never been evaluated.

Therefore, we aimed to evaluate the validity and reproducibility of food group intakes derived from the TMM-FFQ, which includes the response option “constitutionally unable to eat or drink it.” We hope that the findings will contribute to the development of personalized healthcare and medicine.

## METHODS

### Study population

The study was conducted in Sendai, Iwanuma, and Ishinomaki in Miyagi Prefecture, Japan. The ToMMo established seven community support centers in Miyagi Prefecture as local facilities for voluntary admission-type recruitment and health assessment of cohort participants.^4^ The eligibility criteria of the present study were residents aged 20 years or over residing in Miyagi Prefecture and able to visit either Sendai, Iwanuma, or Ishinomaki community support centers. The ToMMo study office recruited 228 men and women who voluntarily agreed to participate in the study; these individuals included both participants and non-participants of the TMM CommCohort Study and the TMM BirThree Cohort Study. All participants provided written informed consent to participate at the study orientation. Among them, 15 participants failed to complete the study owing to a busy schedule or other difficulties. A final total of 89 men and 124 women who completed the study were included in the validity and reproducibility analyses. The protocol of the present study was reviewed and approved by the ethics committee of ToMMo (2019-4-027) and all other collaborating research institutions.

### Data collection

**Figure 1.**
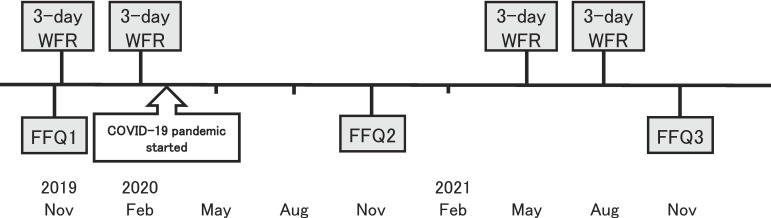
Schedule for the present study. FFQ, food frequency questionnaire; FFQ1, the first FFQ; FFQ2, the second FFQ; FFQ3, the third FFQ; WFR, weighed food record.

Figure 1 shows the study schedule. Between November 2019 and August 2021, participants completed weighed food records (WFRs) for 3 consecutive days four times, once per season; namely, November 2019 (autumn), February 2020 (winter), May 2021 (spring), and August 2021 (summer). We initially planned to ask participants to complete the third 3-day WFR in May 2020 and the fourth 3-day WFR in August 2020. However, we had to interrupt the study owing to the occurrence of the coronavirus disease 2019 (COVID-19) pandemic. After a 1-year interruption, we restarted the study in May 2021. The TMM-FFQ was administered three times at an interval of 1 year; namely, November 2019 (FFQ1), November 2020 (FFQ2), and November 2021 (FFQ3). We initially planned to ask participants to complete the FFQ twice, in November 2019 and in November 2020. However, because of the COVID-19 pandemic, we had to change this plan. Participants were requested to answer FFQ1 approximately 1 week before the start of the food recording period and submit FFQ1 to the center personnel when they visited the community support center. Participants sent FFQ2 to the ToMMo administration office by mail owing to the COVID-19 pandemic. Participants submitted FFQ3 to the center personnel when they visited the community support center. Information on height, weight, smoking status, alcohol drinking, medication, and supplement use were collected using a self-administered questionnaire integrated with the FFQ.

### Reference method

Each consecutive 3-day period consisted of 2 weekdays and 1 weekend day. At the study orientation, participants received guidance from trained dietitians on how to complete the WFRs. Food portions were measured by each participant during meal preparation using supplied portable digital scales (Tanita Co. Ltd., Tokyo, Japan) and measuring spoons and cups. For foods purchased or consumed outside the home, participants were instructed to record the approximate quantity of all foods in the meal and/or the names of the product and company. Trained personnel including dietitians checked food records with participants on site in each community support center the day after each of the 3-day WFR, and foods and weights were coded based on the Standard Tables of Food Composition in Japan 2010.^20^ At the third and fourth 3-day WFR, participants who were anxious about COVID-19 infection submitted the 3-day WFRs via fax or mail to the study office and their responses were checked online.

### FFQ

The TMM-FFQ consists of 139 food and beverage items rated on 10 consumption frequency categories and 3 portion size categories. It asks respondents about their usual consumption of the listed foods during the previous year. The food list was initially developed and used for the Japan Public Health Center-based Prospective Study (JPHC Study),^21^^,^^22^ and then subsequently modified; 12 foods mainly consumed in specific areas (Okinawa and Nagano) or at specific times were excluded and 12 foods consumed throughout the year in urban areas were added.^23^ Shochu highball was also added. The 10 consumption frequency categories for foods are as follows: constitutionally unable to eat it, <once/month or never, 1–3 times/month, 1–2 times/week, 3–4 times/week, 5–6 times/week, once/day, 2–3 times/day, 4–6 times/day, and ≥7 times/day. Portion size is specified for each food item using three standard sizes: small (50% smaller), medium (the standard amount), and large (50% larger). The 10 consumption frequency categories for drinks are as follows: constitutionally unable to drink it, <once/week or never, 1–2 times/week, 3–4 times/week, 5–6 times/week, 1 cup/day, 2–3 cups/day, 4–6 cups/day, 7–9 cups/day, and ≥10 cups/day. If personnel identified missing answers or logical errors in the FFQ responses, participants were asked to provide that information again.

Energy intake was calculated using the Standard Tables of Food Composition in Japan 2010.^20^ Individual food items were categorized into 21 predefined food groups mainly according to the Standard Tables of Food Composition in Japan 2010.^20^ Umeboshi (pickled plum) was included in pickled vegetables, and not in total vegetables but rather in fruits.

### Statistical analysis

The mean intakes of each food group, estimated using the FFQ3, were compared with intakes estimated using the 12-day WFR. Percentage differences were calculated for each food group by dividing the difference in mean intake on the FFQ3 from that on the 12-day WFR by that on the 12-day WFR. To determine the validity of the FFQ, Spearman’s rank correlation coefficients (CCs) for the correlation between food group intakes assessed using the 12-day WFR and those assessed using the FFQ3 were calculated for crude and deattenuated values. The observed CCs were corrected for the attenuating effect of random intra-individual error from the usual intake of food groups. The deattenuated value was calculated using the ratios of the within-individual to between-individual variances based on the 12-day WFR according to the following formula:

$$\text{Deattenuated CC}{}_{\text{x}}=\text{observed CC}{}_{\text{x}}*\text{SQRT(}1+\lambda_{\text{x}}\text{/n)},$$

where the observed CCx is the correlation in energy-adjusted values for food group x, λ_x_ is the ratio of within-individual to between-individual variance, and *n* is the number of WFR.^1^ Energy-adjusted values were calculated using the density method, in which food group intake was divided by total energy intake and expressed as intake per 1,000 kcal.^1^ To measure the validity of the categorization, we calculated the number of participants classified into the same, adjacent, and extreme categories by joint classification according to both quintiles using the 12-day WFR and the FFQ3. We also conducted similar analyses using the FFQ1 instead of the FFQ3.

Percentage differences were also calculated for each food group by dividing the difference in mean intake on the FFQ3 from that on the FFQ1 by that on the FFQ1. To determine the reproducibility of the FFQ, Spearman’s rank CC_x_ was calculated to examine the correlation between food group intakes assessed using the FFQ1 and FFQ3. We also calculated the number of participants classified into the same, adjacent, and extreme categories by joint classification according to both quintiles using the FFQ1 and FFQ3.

All analyses were conducted using SAS version 9.4 software (SAS Institute Inc., Cary, NC, USA).

## RESULTS

Table 1 shows the characteristics of the participants. The mean age was 57.2 (standard deviation [SD], 16.2) years in men and 53.8 (SD, 15.5) years in women. Body mass index was 24.1 (SD, 3.0) kg/m^2^ in men and 22.7 (SD, 3.9) kg/m^2^ in women. Current smokers accounted for 20.2% of men and 0.8% of women. Current drinkers accounted for 71.9% of men and 40.3% of women.

**Table 1.** Characteristics of participants

	Men		Women	
Age, years, mean (SD)	57.2	(16.2)	53.8	(15.5)
Height, cm, mean (SD)	167.4	(6.5)	156.9	(5.5)
Weight, kg, mean (SD)	67.7	(9.7)	55.8	(9.9)
Body mass index, kg/m^2^, mean (SD)	24.1	(3.0)	22.7	(3.9)
Smoking status, *n* (%)				
Current smoker	18	(20.2)	1	(0.8)
Past smoker	44	(49.5)	25	(20.2)
Never smoker	27	(30.3)	98	(79.0)
Alcohol drinking, *n* (%)				
Current drinker	64	(71.9)	50	(40.3)
Past drinker	3	(3.4)	1	(0.8)
Never drinker	17	(19.1)	58	(46.8)
Constitutionally never drinker	5	(5.6)	15	(12.1)

SD, standard deviation.

Table 2 shows the percentages of participants who chose the response option “constitutionally unable to eat or drink it” for each food item. The median percentages were 0.0% among men and 0.8% among women. The percentages were over 5% for soy milk (5.6%), papaya (5.6%), pork liver (12.4%), chicken liver (10.1%), and alcoholic beverages (5.6%) in men, and papaya (8.1%), eel (7.3%), pork liver (8.9%), chicken liver (12.1%), low-fat milk (6.5%), margarine for spread (6.5%), alcoholic beverages (12.1%), and canned coffee (8.9%) in women.

**Table 2.** The percentages of participants who chose the response option “constitutionally unable to eat or drink it” for each food item

	Men	Women
	%	%
Cereals		
Breads	0.0	0.0
Udon (thick wheat noodles)	0.0	0.0
Soba (buckwheat noodles)	0.0	0.0
Ramen (Chinese noodles)	0.0	0.0
Rice cake	0.0	0.8
Potatoes and starches		
Sweet potato	0.0	0.0
Potato	0.0	0.0
Taro	2.3	0.8
Konjac	0.0	0.0
Pulses		
Tofu for miso soup	0.0	0.0
Tofu for other dishes	0.0	0.0
Kori-dofu (freeze dried tofu)	0.0	0.0
Nama-age (fried slices of drained tofu)	0.0	0.0
Abura-age (fried thin slices of pressed tofu)	0.0	0.0
Natto (fermented soybeans)	1.1	0.0
Soy milk	5.6	3.2
Nuts and seeds		
Peanuts	0.0	1.6
Vegetables		
Carrot	1.1	0.0
Spinach	1.1	1.6
Pumpkin	0.0	0.0
Cabbage	0.0	0.0
Japanese radish	0.0	0.0
Takuan-zuke (pickled with rice bran and salt)	0.0	3.2
Pickled green leafy vegetables	1.1	4.1
Pickled Chinese cabbage	1.1	2.4
Pickled cucumber	2.3	3.2
Pickled eggplant	3.4	4.0
Sweet pepper	0.0	0.8
Tomato	0.0	0.8
Welsh onion	0.0	0.0
Chinese chive	0.0	0.0
Garland chrysanthemum	2.3	4.0
Komatsuna (spinach mustard)	0.0	0.0
Broccoli	1.1	0.0
Onion	0.0	0.0
Cucumber	1.1	0.0
Eggplant	4.5	0.0
Chinese cabbage	0.0	0.0
Edible burdock	0.0	0.0
Bean sprout	0.0	0.0
Sayaingen (young kidney beans pods)	1.1	0.0
Lettuce	0.0	0.0
Tomato juice	3.4	1.6
Fruits		
Papaya	5.6	8.1
Mandarin	0.0	0.0
Other citrus fruits	0.0	0.0
Apple	0.0	0.0
Japanese persimmon	3.4	2.4
Strawberry	0.0	0.8
Grapes	0.0	0.0
Melon	0.0	4.0
Watermelon	1.1	0.8
Peach	1.1	0.8
Pear	0.0	0.0
Kiwifruit	2.3	1.6
Pineapple	1.1	0.8
Banana	0.0	3.2
Umeboshi (pickled plum)	4.5	3.2
Marmalade	4.5	2.4
100% orange juice beverage	0.0	0.0
100% apple juice beverage	0.0	0.0
Fungi		
Shiitake	1.1	0.8
Enokitake	1.1	0.8
Shimeji	1.1	0.8
Algae		
Wakame, kombu	0.0	0.0
Hijiki	0.0	0.0
Nori	0.0	0.0
Fish and shellfish		
Salted fish (Pacific cod, atka mackerel, salmon)	2.3	0.8
Dried fish	2.3	1.6
Canned tuna	3.4	0.0
Salmon, trout	2.3	0.8
Bonito, tuna	0.0	0.0
Japanese amberjack	3.4	2.4
Cod, flatfish	1.1	1.6
Sea bream	3.4	4.8
Horse mackerel, sardine	4.5	1.6
Pacific saury, mackerel	2.3	1.6
Shirasuboshi (boiled and dried whitebait)	4.5	1.6
Cod roe, salmon roe	2.3	1.6
Eel	4.5	7.3
Squid	2.3	1.6
Octopus	2.3	1.6
Shrimp	1.1	0.0
Short-neck clam, freshwater clam	1.1	0.0
Chikuwa (fish paste product)	0.0	0.0
Kamaboko (fish paste product)	0.0	0.0
Meats		
Beef, steak	0.0	1.6
Beef, grilled	0.0	1.6
Beef, stir-fried	0.0	1.6
Beef, stewed	0.0	1.6
Pork, stir-fried	0.0	0.8
Pork, deep-fried	0.0	1.6
Pork, stewed	0.0	0.8
Pork, simmered	1.1	0.8
Pork, in soup	0.0	0.8
Pork liver	12.4	8.9
Chicken, grilled	0.0	1.6
Chicken, stir-fried	0.0	0.8
Chicken, simmered	0.0	0.8
Chicken, deep-fried	0.0	1.6
Chicken liver	10.1	12.1
Loin ham	1.1	0.8
Sausage	0.0	0.8
Bacon	2.3	1.6
Eggs		
Egg	0.0	0.0
Milk and dairy products		
Low-fat milk	2.3	6.5
Milk	2.3	3.2
Cheese	0.0	0.0
Yogurt	0.0	0.8
Lactic acid bacteria beverage	0.0	1.6
Fats and oils		
Butter for spread	2.3	1.6
Margarine for spread	4.5	6.5
Confectionaries		
Japanese traditional confectionaries	1.1	0.0
Cake	1.1	0.8
Biscuit, cookie	0.0	0.0
Chocolate	0.0	0.0
Alcoholic beverages	5.6	12.1
Nonalcoholic beverages		
Green tea (sencha)	0.0	0.8
Green tea (bancha, genmaicha)	0.0	0.0
Oolong tea	0.0	1.6
Black tea	1.1	0.0
Coffee (not canned coffee)	0.0	4.0
Canned coffee	1.1	8.9
Soup	0.0	0.0
Soft drink	1.1	1.6
Energy drink	2.3	3.2
Seasonings and spices		
Dressing	0.0	0.0
Mayonnaise	1.1	0.0
Worcester sauce	0.0	0.8
Ketchup	0.0	0.0

**Median**	**0.0**	**0.8**

Table 3 shows daily intakes for 21 food groups assessed using the 12-day WFR and FFQ3, their percentage differences, and their correlations. The percentage difference in intakes between the FFQ3 and 12-day WFR varied from −81.3% for seasonings and spices to 97.8% for milk and dairy products in men. The deattenuated CCs varied from 0.23 for fats and oils to 0.89 for alcoholic beverages; the median across 21 food groups was 0.49 in men. The percentage difference in intakes between the FFQ3 and 12-day WFR varied from −88.2% for sugar to 61.7% for milk and dairy products in women. The deattenuated CCs varied from 0.14 for algae to 0.70 for fish and shellfish; the median across 21 food groups was 0.45 in women. Medians of deattenuated CCs for the correlation between intakes assessed using the 12-day WFR and FFQ1 were 0.55 in men and 0.41 in women (eTable 1).

**Table 3.** Food group intakes assessed using the 12-day WFR and FFQ3, their percentage differences, and their correlations

	Men							Women						
	
	12-day WFR		FFQ3		%^a^	CC		12-day WFR		FFQ3		%^a^	CC	
	
	Mean (SD)		Mean (SD)			Crude^b^	Deattenuated^c^	Mean (SD)		Mean (SD)			Crude^b^	Deattenuated^c^
	g		g					g		g				
Cereals	459.7	(153.9)	478.5	(159.1)	4.1	0.62	0.64	325.5	(91.7)	382.2	(130.2)	17.4	0.51	0.66
Potatoes and starches	36.7	(21.1)	27.2	(20.7)	−25.9	0.27	0.33	39.5	(22.2)	30.7	(21.4)	−22.2	0.15	0.23
Sugar	7.7	(5.3)	1.5	(4.4)	−80.9	0.23	0.27	7.1	(5.3)	0.8	(2.9)	−88.2	0.17	0.19
Pulses	62.2	(42.7)	79.7	(93.3)	28.0	0.50	0.46	67.6	(46.5)	95.2	(102.5)	40.9	0.48	0.53
Nuts and seeds	3.5	(5.0)	5.9	(19.4)	65.6	0.42	0.43	3.5	(4.2)	3.0	(5.1)	−14.2	0.26	0.29
Vegetables	292.1	(147.0)	169.0	(149.1)	−42.2	0.60	0.60	276.9	(140.8)	204.7	(159.4)	−26.1	0.44	0.48
Green and yellow vegetables	100.5	(70.9)	65.7	(71.7)	−34.7	0.60	0.63	96.8	(65.0)	84.7	(81.7)	−12.4	0.42	0.45
White vegetables	191.6	(92.2)	103.3	(84.4)	−46.1	0.52	0.49	180.2	(97.7)	120.0	(90.2)	−33.4	0.39	0.36
Pickled vegetables	12.9	(15.5)	12.2	(17.7)	−5.3	0.61	0.67	10.1	(10.9)	12.3	(20.6)	22.0	0.48	0.58
Fruits	94.1	(81.8)	152.3	(260.4)	61.8	0.65	0.65	94.0	(75.8)	142.9	(154.8)	52.0	0.49	0.49
Fungi	14.2	(11.3)	11.3	(12.2)	−20.3	0.63	0.72	14.2	(9.7)	13.7	(11.9)	−2.9	0.48	0.56
Algae	7.2	(6.9)	7.6	(7.9)	6.2	0.40	0.35	7.1	(8.0)	8.2	(9.6)	15.2	0.17	0.14
Fish and shellfish	74.0	(48.4)	61.2	(46.6)	−17.3	0.52	0.49	61.5	(40.0)	55.1	(46.4)	−10.4	0.60	0.70
Meats	101.0	(44.9)	84.6	(49.0)	−16.2	0.38	0.48	80.4	(32.2)	71.1	(50.2)	−11.6	0.43	0.53
Eggs	39.9	(22.7)	38.9	(40.6)	−2.7	0.50	0.58	35.5	(18.5)	44.6	(65.7)	25.7	0.47	0.44
Milk and dairy products	132.4	(103.3)	261.9	(512.9)	97.8	0.74	0.75	142.4	(91.5)	230.2	(275.5)	61.7	0.51	0.59
Fats and oils	13.7	(5.7)	11.6	(6.1)	−15.7	0.18	0.23	11.8	(4.5)	11.7	(8.1)	−1.0	0.21	0.21
Confectionaries	35.2	(29.7)	22.3	(43.2)	−36.5	0.32	0.32	42.2	(25.4)	29.0	(34.3)	−31.2	0.29	0.35
Alcoholic beverages	221.8	(292.9)	247.1	(376.3)	11.4	0.87	0.89	90.3	(217.7)	100.9	(333.9)	11.8	0.65	0.66
Nonalcoholic beverages	569.7	(295.8)	612.7	(578.6)	7.5	0.39	0.37	602.5	(292.3)	602.9	(512.2)	0.1	0.20	0.23
Seasonings and spices	120.5	(52.5)	22.5	(13.3)	−81.3	0.22	0.25	105.8	(49.3)	22.5	(15.5)	−78.7	0.38	0.43

**Median**						**0.50**	**0.49**						**0.43**	**0.45**

CC, correlation coefficient; FFQ, food frequency questionnaire; FFQ3, the third FFQ; SD, standard deviation; WFR, weighed food record.

^a^Percentage differences: (FFQ3 − 12-day WFR)/12-day WFR × 100 (%).

^b^Spearman’s rank CC based on crude values.

^c^Spearman’s rank CC based on energy-adjusted values and expressed as deattenuated CC. Deattenuated CCx = observed CCx * SQRT (1 + λx/n), where λx is the ratio of within- to between-individual variance for food group x, and *n* is the number of WFR.

Table 4 shows a comparison of the 12-day WFR and the FFQ3 for energy-adjusted food group intakes using joint classification by quintiles. The proportion of participants classified into the opposite extreme category was ≤5% for most food groups. Classification into the same and adjacent categories for food groups ranged from 56.2% for fats and oils and seasonings and spices to 93.3% for alcoholic beverages in men, and from 56.5% for potatoes and starches to 76.6% for fish and shellfish in women. The median values for the same and adjacent categories were 73.0% in men and 66.9% in women. Comparison of the 12-day WFR and the FFQ1 responses showed that the median values for the same and adjacent categories were 73.0% in men and 65.3% in women (eTable 2).

**Table 4.** Comparison of the 12-day WFR and the FFQ3 for energy-adjusted food group intakes based on joint classification by quintiles

	Men			Women		
	
	Same category	Same and adjacent category	Extreme category	Same category	Same and adjacent category	Extreme category
	%	%	%	%	%	%
Cereals	30.3	77.5	1.1	37.9	75.8	1.6
Potatoes and starches	30.4	59.6	2.2	21.0	56.5	4.8
Sugar^a^	—	—	—	—	—	—
Pulses	34.8	71.9	3.4	25.8	72.6	3.2
Nuts and seeds^a^	—	—	—	28.2	57.3	6.5
Vegetables	42.7	77.5	3.4	28.2	73.4	3.2
Green and yellow vegetables	40.5	77.5	1.1	28.2	71.0	0.8
White vegetables	27.0	77.5	3.4	34.7	65.3	4.0
Pickled vegetables	40.5	73.0	1.1	37.1	71.8	2.4
Fruits	34.8	80.9	0.0	37.1	64.5	1.6
Fungi	42.7	80.9	2.3	29.0	74.2	2.4
Algae	28.1	58.4	3.4	26.6	58.1	4.8
Fish and shellfish	34.8	61.8	1.1	35.5	76.6	0.0
Meats	23.6	66.3	3.4	26.6	66.9	0.8
Eggs	36.0	74.2	2.2	30.7	62.9	3.2
Milk and dairy products	42.7	83.1	0.0	38.7	75.0	2.4
Fats and oils	24.7	56.2	3.4	24.2	59.7	4.8
Confectionaries	30.3	61.8	4.5	22.6	59.7	1.6
Alcoholic beverages^a^	66.3	93.3	0.0	—	—	—
Nonalcoholic beverages	27.0	67.4	4.5	23.4	59.7	4.0
Seasonings and spices	24.7	56.2	4.5	27.4	67.7	2.4

**Median**	**34.8**	**73.0**	**2.3**	**28.2**	**66.9**	**2.4**

FFQ, food frequency questionnaire; FFQ3, the third FFQ; WFR, weighed food record.

^a^Intakes of sugar, nuts and seeds (only for men), and alcoholic beverages (only for women) were not categorized into quintiles, because the percentages of participants who reported no consumption of these foods were high.

Table 5 shows the daily intakes for 21 food groups assessed using the FFQ1 and FFQ3, their percentage differences, and their correlations. The percentage difference in intakes between the FFQ3 and FFQ1 varied from −33.7% for sugar to 105.7% for nuts and seeds in men. The energy-adjusted CCs varied from 0.43 for potatoes and starches and fats and oils to 0.86 for alcoholic beverages; the median across 21 food groups was 0.60 in men. The percentage difference in intakes between the FFQ3 and FFQ1 varied from −25.3% for potatoes and starches to 16.7% for eggs in women. The energy-adjusted CCs varied from 0.36 for potatoes and starches to 0.77 for alcoholic beverages; the median across 21 food groups was 0.51 in women.

**Table 5.** Food group intakes assessed using the FFQ1 and FFQ3, their percentage differences, and their correlations

	Men							Women						
	
	FFQ1		FFQ3		%^a^	CC		FFQ1		FFQ3		%^a^	CC	
	
	Mean (SD)		Mean (SD)			Crude^b^	Energy- adjusted^c^	Mean (SD)		Mean (SD)			Crude^b^	Energy- adjusted^c^
	g		g					g		g				
Cereals	536.0	(191.8)	478.5	(159.1)	−10.7	0.60	0.56	403.7	(131.6)	382.2	(130.2)	−5.3	0.46	0.57
Potatoes and starches	31.0	(30.0)	27.2	(20.7)	−12.2	0.47	0.43	41.1	(68.8)	30.7	(21.4)	−25.3	0.39	0.36
Sugar	2.2	(7.1)	1.5	(4.4)	−33.7	0.71	0.72	0.8	(2.1)	0.8	(2.9)	7.4	0.57	0.57
Pulses	67.8	(64.0)	79.7	(93.3)	17.5	0.66	0.62	102.6	(138.7)	95.2	(102.5)	−7.2	0.48	0.49
Nuts and seeds	2.9	(5.3)	5.9	(19.4)	105.7	0.65	0.67	2.8	(5.2)	3.0	(5.1)	6.8	0.54	0.51
Vegetables	179.1	(128.2)	169.0	(149.1)	−5.6	0.64	0.60	223.7	(190.4)	204.7	(159.4)	−8.5	0.58	0.47
Green and yellow vegetables	68.1	(62.8)	65.7	(71.7)	−3.6	0.56	0.51	99.3	(158.5)	84.7	(81.7)	−14.7	0.56	0.47
White vegetables	111.0	(81.3)	103.3	(84.4)	−6.9	0.64	0.64	124.4	(68.8)	120.0	(90.2)	−3.5	0.56	0.45
Pickled vegetables	11.8	(18.8)	12.2	(17.7)	3.5	0.70	0.74	11.6	(16.3)	12.3	(20.6)	5.5	0.70	0.70
Fruits	132.9	(129.2)	152.3	(260.4)	14.6	0.77	0.76	152.9	(187.2)	142.9	(154.8)	−6.5	0.64	0.65
Fungi	10.7	(12.0)	11.3	(12.2)	6.0	0.73	0.68	15.6	(28.1)	13.7	(11.9)	−11.9	0.59	0.58
Algae	7.2	(7.0)	7.6	(7.9)	6.2	0.63	0.55	8.5	(11.4)	8.2	(9.6)	−3.8	0.51	0.48
Fish and shellfish	64.9	(57.6)	61.2	(46.6)	−5.8	0.59	0.55	59.5	(53.7)	55.1	(46.4)	−7.3	0.60	0.55
Meats	97.5	(92.9)	84.6	(49.0)	−13.2	0.51	0.45	86.6	(75.5)	71.1	(50.2)	−17.8	0.51	0.43
Eggs	41.4	(51.0)	38.9	(40.6)	−6.1	0.65	0.56	38.3	(43.1)	44.6	(65.7)	16.7	0.56	0.44
Milk and dairy products	211.1	(249.2)	261.9	(512.9)	24.0	0.58	0.54	256.0	(345.7)	230.2	(275.5)	−10.1	0.58	0.56
Fats and oils	13.0	(8.1)	11.6	(6.1)	−10.9	0.53	0.43	12.2	(5.9)	11.7	(8.1)	−4.3	0.60	0.42
Confectionaries	18.1	(29.0)	22.3	(43.2)	23.2	0.58	0.58	30.1	(34.1)	29.0	(34.3)	−3.4	0.52	0.45
Alcoholic beverages	221.4	(288.5)	247.1	(376.3)	11.6	0.88	0.86	106.6	(255.5)	100.9	(333.9)	−5.3	0.79	0.77
Nonalcoholic beverages	643.4	(549.5)	612.7	(578.6)	−4.8	0.62	0.65	589.8	(442.7)	602.9	(512.2)	2.2	0.54	0.58
Seasonings and spices	24.7	(12.6)	22.5	(13.3)	−8.8	0.64	0.61	23.7	(13.3)	22.5	(15.5)	−4.9	0.65	0.55

**Median**						**0.64**	**0.60**						**0.56**	**0.51**

CC, correlation coefficient; FFQ, food frequency questionnaire; FFQ1, the first FFQ; FFQ3, the third FFQ; SD, standard deviation.

^a^Percentage differences: (FFQ3 − FFQ1)/FFQ1 × 100 (%).

^b^Spearman’s rank CC based on crude values.

^c^Spearman’s rank CC based on energy-adjusted values.

Table 6 shows the comparison of the FFQ1 and the FFQ3 for energy-adjusted food group intake based on joint classification by quintiles. The proportion of participants classified into the opposite extreme category was ≤5% for all food groups. The median values of the same and adjacent categories were 77.5% in men and 73.8% in women.

**Table 6.** Comparison of the FFQ1 and the FFQ3 for energy-adjusted food group intakes based on joint classification by quintiles

	Men			Women		
	
	Same category	Same and adjacent category	Extreme category	Same category	Same and adjacent category	Extreme category
	%	%	%	%	%	%
Cereals	32.6	74.2	2.3	44.4	75.8	1.6
Potatoes and starches	32.6	67.4	2.2	28.2	67.8	2.4
Sugar^a^	—	—	—	—	—	—
Pulses	36.0	79.8	1.1	37.1	74.2	0.8
Nuts and seeds^a^	—	—	—	—	—	—
Vegetables	37.1	77.5	1.1	37.1	69.4	3.2
Green and yellow vegetables	40.5	74.2	4.5	34.7	73.4	2.4
White vegetables	37.1	80.9	0.0	32.3	74.2	3.2
Pickled vegetables	51.7	84.3	0.0	44.4	78.2	0.0
Fruits	49.5	86.5	1.1	34.7	80.7	1.6
Fungi	45.0	83.2	0.0	41.9	75.8	1.6
Algae	33.7	74.1	0.0	37.9	71.8	4.0
Fish and shellfish	34.8	79.8	2.2	32.3	71.8	1.6
Meats	28.1	73.0	1.1	36.3	72.6	2.4
Eggs	38.2	80.9	2.2	33.1	68.6	4.0
Milk and dairy products	40.5	76.4	1.1	33.1	76.6	2.4
Fats and oils	30.3	69.7	2.3	29.8	66.9	0.8
Confectionaries	41.6	74.2	3.4	29.0	69.4	3.2
Alcoholic beverages^a^	66.3	93.3	1.1	—	—	—
Nonalcoholic beverages	40.5	77.5	0.0	40.3	75.0	2.4
Seasonings and spices	33.7	77.5	0.0	33.9	74.2	2.4

**Median**	**37.1**	**77.5**	**1.1**	**34.7**	**73.8**	**2.4**

FFQ, food frequency questionnaire; FFQ1, the first FFQ; FFQ3, the third FFQ.

^a^Intakes of sugar, nuts and seeds, and alcoholic beverages (only for women) were not categorized into quintiles, because the percentages of participants who reported no consumption of these foods were high.

## DISCUSSION

The present study examined the validity and reproducibility of food group intakes derived from the TMM-FFQ developed for genomic and omics research and containing the response option “constitutionally unable to eat or drink it.” The percentages of participants who chose this response option were not negligible for some food groups, although the total percentages were low. The validity of the TMM-FFQ compared with 12-day WFR and the reproducibility of the TMM-FFQ were reasonable for the 21 selected food groups. The agreement rates based on cross-classification by quintiles for validity and reproducibility were also acceptable.

In the validity analysis, the median CC across 21 food groups was 0.49 in men and 0.45 in women. CC values were >0.40 for 14 food groups in men and 13 food groups in women, showing that the TMM-FFQ has reasonable ranking ability. In the JPHC Study Cohort I, the median energy-adjusted CC of 19 food groups was 0.38 in men and 0.32 in women.^24^ In the JPHC Study Cohort II, the median energy-adjusted CC of 19 food groups was 0.41 in men and 0.30 in women.^25^ The TMM-FFQ was developed by modifying some food items,^23^ and by adding the response option “constitutionally unable to eat or drink it” for individual food and drink items from the JPHC-FFQ. These results suggest that the validity of the FFQ was retained even after adding the response option “constitutionally unable to eat or drink it.” The findings also suggest that the TMM-FFQ could help to clarify the association between diet and disease, even though the percentages of participants who chose this response option were not negligible for some food groups.

The TMM-FFQ assessed the usual consumption of listed foods during the previous year; therefore, the FFQ3 assessed the usual consumption of listed foods from November 2020 to November 2021. The 12-day WFR was conducted in November 2019, February 2020, May 2021, and August 2021 because of schedule changes owing to the COVID-19 pandemic. This means that the administration period of the TMM-FFQ did not fully overlap with that of the WFR. However, we have confirmed that the validity of the FFQ1 when compared with the 12-day WFR was similar to that of the FFQ3 (as shown in eTable 1 and eTable 2).

The deattenuated CCs for the correlation between intakes assessed using the TMM-FFQ and 12-day WFR varied from 0.23 for fats and oils to 0.89 for alcoholic beverages in men and from 0.14 for algae to 0.70 for fish and shellfish in women. One review of FFQs developed in Japan noted considerable variation in the correlations between intakes assessed using WFRs and FFQs among food groups; the CCs ranged from 0.15 for potatoes to 0.77 for bread.^3^ Variation may partly reflect differences in the number of food items, ability to recall intake frequencies and portion sizes, the wording of FFQ questions, and between-person variation in the consumption of different food groups.^3^ The same review showed that the validity for reported consumption of a food group was high for rice, bread, milk or milk plus dairy products, fruits, and alcoholic beverages, and was low for potatoes and algae.^3^ Another recent review of FFQs developed in Japan showed that the validity for reported consumption of a food group was high for fruit and milk.^2^ The present study showed similar results: the validity for reported consumption of a food group was high for cereals, milk and dairy products, fruits, and alcoholic beverages, and was low for potatoes and starches and algae. Within the milk and dairy products and fruits food groups, low-fat milk and papaya, respectively, had non-negligible percentages of “constitutionally unable to eat or drink it” responses, and the percentage of these responses was high for alcoholic beverages. These findings suggest that this response option did not affect the validity of the FFQ. Differences in the validity of food groups in FFQs should be considered when comparing health effects among food groups.

The reproducibility analysis showed that the median CC across 21 food groups was 0.60 in men and 0.51 in women. CC values were >0.50 for 18 food groups in men and 11 food groups in women, indicating that the TMM-FFQ has reasonable reproducibility. In the JPHC Study Cohort I, the median energy-adjusted CC of food groups was 0.50 in men and 0.49 in women.^26^ In the JPHC Study Cohort II, the median energy-adjusted CC of food groups was 0.54 in men and 0.50 in women.^25^ These findings suggest that the reproducibility of the TMM-FFQ is comparable to that of the JPHC-FFQ.

Real changes in regular dietary intake and random variation in FFQ responses are considered factors that affect the reproducibility of FFQs. One review demonstrated that FFQs developed in Japan were moderately reproducible at intervals between 9 months and 1 year.^3^ One meta-analysis also demonstrated that the reproducibility was higher when FFQs were administered over a short period (≤6 months) compared with a long period (>6 months), suggesting that a shorter interval between repeated FFQ administrations is a key factor contributing to high reproducibility of FFQs.^27^ The TMM-FFQ asks respondents about their usual consumption of listed foods during the previous year. However, the interval between the two FFQs in this study was 2 years, because FFQ1 was conducted in November 2019 and FFQ3 was conducted in November 2021 owing to the COVID-19 pandemic. There is some evidence that the COVID-19 pandemic has affected some aspects of people’s lives, including dietary habits.^28^^,^^29^ Despite the long interval between FFQ administration and possible lifestyle changes during the COVID-19 pandemic, the reproducibility of the TMM-FFQ has been demonstrated.

The present study had several limitations. First, we assumed that food group intakes derived from the WFR were the gold standard, and our assessment of relative validity depended on the extent to which WFRs are accurate. However, WFRs are susceptible to measurement error owing to erroneous recording and potential changes in dietary behavior. Nevertheless, compared with 24-hour dietary recall or other instruments that rely on memory, WFR errors are considered to have a low correlation with FFQ errors.^1^ Moreover, biomarkers cannot be used to examine the validity of most food group intakes. Second, the generalizability of the present results is limited because participants were not randomly selected from the general Japanese population. In addition, participants may have been highly health conscious, as all of them completed the study despite its strict design. This may have led to overestimation of the validity and reproducibility of the TMM-FFQ. Third, some participants completed their responses with the help of the individuals who usually prepared their meals; the questionnaire’s validity and reproducibility may have been slightly different if these participants had completed the questionnaires by themselves. Obtaining cooperation from individuals who usually prepare meals for survey participants may be necessary when using the FFQ. Finally, the reason why participants selected the response option “constitutionally unable to eat or drink it” is unclear. Some may have chosen this option because of genetically-based food allergies or preferences, whereas others may have chosen the option because of taste preferences. However, participants were instructed to choose the “<once or never” option if they did not consume specific foods and drinks for reasons other than constitutional ones.

In conclusion, this study demonstrated the validity and reproducibility of the TMM-FFQ with the response option “constitutionally unable to eat or drink it” for food group intakes among community-dwelling Japanese adults, although the percentages of participants who chose this response option were not negligible for some food groups. This finding suggests that the TMM-FFQ is suitable for the assessment of food group intakes in the TMM cohort studies.

## SUPPLEMENTARY MATERIAL

eTable 1. Food group intakes assessed using the 12-day WFR and FFQ1, their percentage differences, and their correlations

eTable 2. Comparison of the 12-day WFR and the FFQ1 for energy-adjusted food group intakes based on joint classification by quintiles
