# Supplementary material for: Validity and Reproducibility of Food Group Intakes in a Self-administered Food Frequency Questionnaire for Genomic and Omics Research: The Tohoku Medical Megabank Project
Source: J Epidemiol. 2025 Mar 5;35(3):109–17. doi: 10.2188/jea.JE20240064 (PMC11821377; doi:10.2188/jea.JE20240064)
Supplement: Supplementary file 1 [file je-35-109-s001.pdf]

eTable 1. Food group intakes assessed using the 12-day WFR and FFQ1, their percentage differences, and their correlations

	Men							Women						
	12-day WFR Mean (SD) g		FFQ1 Mean (SD) g		%[a]	CC		12-day WFR Mean (SD) g		FFQ1 Mean (SD) g		%[a]	CC	
						Crude[b]	Deattenuated[c]						Crude[b]	Deattenuated[c]
Cereals	459.7	(153.9)	536.0	(191.8)	16.6	0.59	0.57	325.5	(91.7)	403.7	(131.6)	24.0	0.45	0.59
Potatoes and starches	36.7	(21.1)	31.0	(30.0)	-15.6	0.36	0.37	39.5	(22.2)	41.1	(68.8)	4.0	0.37	0.48
Sugar	7.7	(5.3)	2.2	(7.1)	-71.2	0.14	0.15	7.1	(5.3)	0.8	(2.1)	-89.0	0.30	0.33
Pulses	62.2	(42.7)	67.8	(64.0)	8.9	0.64	0.74	67.6	(46.5)	102.6	(138.7)	51.8	0.41	0.42
Nuts and seeds	3.5	(5.0)	2.9	(5.3)	-19.5	0.38	0.38	3.5	(4.2)	2.8	(5.2)	-19.7	0.30	0.32
Vegetables	292.1	(147.0)	179.1	(128.2)	-38.7	0.55	0.55	276.9	(140.8)	223.7	(190.4)	-19.2	0.49	0.41
Green and yellow vegetables	100.5	(70.9)	68.1	(62.8)	-32.2	0.53	0.56	96.8	(65.0)	99.3	(158.5)	2.6	0.40	0.34
White vegetables	191.6	(92.2)	111.0	(81.3)	-42.1	0.52	0.52	180.2	(97.7)	124.4	(68.8)	-31.0	0.42	0.33
Pickled vegetables	12.9	(15.5)	11.8	(18.8)	-8.5	0.53	0.60	10.1	(10.9)	11.6	(16.3)	15.6	0.59	0.68
Fruits	94.1	(81.8)	132.9	(129.2)	41.2	0.61	0.64	94.0	(75.8)	152.9	(187.2)	62.5	0.49	0.49
Fungi	14.2	(11.3)	10.7	(12.0)	-24.8	0.60	0.67	14.2	(9.7)	15.6	(28.1)	10.2	0.53	0.62
Algae	7.2	(6.9)	7.2	(7.0)	-0.008	0.32	0.37	7.1	(8.0)	8.5	(11.4)	19.8	0.28	0.26
Fish and shellfish	74.0	(48.4)	64.9	(57.6)	-12.2	0.60	0.63	61.5	(40.0)	59.5	(53.7)	-3.3	0.58	0.65
Meats	101.0	(44.9)	97.5	(92.9)	-3.5	0.36	0.34	80.4	(32.2)	86.6	(75.5)	7.6	0.34	0.44
Eggs	39.9	(22.7)	41.4	(51.0)	3.6	0.54	0.57	35.5	(18.5)	38.3	(43.1)	7.7	0.42	0.39
Milk and dairy products	132.4	(103.3)	211.1	(249.2)	59.4	0.60	0.66	142.4	(91.5)	256.0	(345.7)	79.8	0.53	0.61
Fats and oils	13.7	(5.7)	13.0	(8.1)	-5.3	0.23	0.31	11.8	(4.5)	12.2	(5.9)	3.5	0.20	0.25
Confectionaries	35.2	(29.7)	18.1	(29.0)	-48.4	0.40	0.43	42.2	(25.4)	30.1	(34.1)	-28.7	0.28	0.29
Alcoholic beverages	221.8	(292.9)	221.4	(288.5)	-0.2	0.82	0.82	90.3	(217.7)	106.6	(255.5)	18.0	0.75	0.77
Nonalcoholic beverages	569.7	(295.8)	643.4	(549.5)	12.9	0.25	0.30	602.5	(292.3)	589.8	(442.7)	-2.1	0.14	0.19
Seasonings and spices	120.5	(52.5)	24.7	(12.6)	-79.5	0.21	0.09	105.8	(49.3)	23.7	(13.3)	-77.6	0.33	0.31
**Median**						**0.53**	**0.55**						**0.41**	**0.41**

CC, correlation coefficient; FFQ, food frequency questionnaire; FFQ1, the first FFQ; SD, standard deviation; WFR, weighed food record.

[a]Percentage differences: (FFQ1-12-day WFR)/12-day WFR×100 (%).

[b]Spearman's rank CC based on crude values.

[c]Spearman's rank CC based on energy-adjusted values and expressed as deattenuated CC. Deattenuated CCx = observed CCx * SQRT (1 + λx/n), where λx is the ratio of within- to between-individual variance for food group x, and n is the number of WFR.

eTable 2. Comparison of the 12-day WFR and the FFQ1 for energy-adjusted food group intakes based on joint classification by quintiles

	Men			Women		
	Same category %	Same and adjacent category %	Extreme category %	Same category %	Same and adjacent category %	Extreme category %
Cereals	37.1	75.3	4.5	29.0	75.0	2.4
Potatoes and starches	20.2	59.5	4.5	32.3	67.8	4.0
Sugar[a]	-	-	-	-	-	-
Pulses	43.8	83.1	1.1	33.9	67.0	3.2
Nuts and seeds[a]	-	-	-	-	-	-
Vegetables	32.6	74.2	2.2	30.7	67.8	2.4
Green and yellow vegetables	38.2	76.4	2.3	29.8	63.7	4.0
White vegetables	29.2	73.0	2.2	21.8	62.9	5.6
Pickled vegetables	30.3	73.0	2.2	38.7	75.8	0.8
Fruits	38.2	78.7	0.0	26.6	66.9	0.8
Fungi	40.5	75.3	1.1	31.5	71.0	0.8
Algae	37.1	61.8	5.6	29.8	61.3	6.5
Fish and shellfish	36.0	73.0	2.2	30.6	71.0	0.0
Meats	27.0	64.0	3.4	29.0	60.5	1.6
Eggs	37.1	71.9	2.3	31.5	63.7	5.7
Milk and dairy products	43.8	79.8	1.1	37.9	73.4	0.8
Fats and oils	23.6	56.2	2.3	26.6	58.1	5.6
Confectionaries	28.1	70.8	3.4	24.2	55.7	3.2
Alcoholic beverages[a]	59.6	89.9	0.0	-	-	-
Nonalcoholic beverages	24.7	62.9	4.5	25.0	62.1	6.5
Seasonings and spices	18.0	49.4	4.5	16.9	57.3	1.6
**Median**	**36.0**	**73.0**	**2.3**	**29.8**	**65.3**	**2.8**

FFQ, food frequency questionnaire; FFQ1, the first FFQ; WFR, weighed food record.

[a]Intakes of sugar, nuts and seeds, and alcoholic beverages (only for women) were not categorized into quintiles, because the percentages of participants who reported no consumption of these foods were high.
